# HIV epidemic, prevention technologies, and the new generations: trends and opportunities for epidemic response

**DOI:** 10.1590/0102-311XEN144223

**Published:** 2023-12-08

**Authors:** Alexandre Grangeiro, Dulce Ferraz, Laio Magno, Eliana Miura Zucchi, Márcia Thereza Couto, Ines Dourado

**Affiliations:** 1 Faculdade de Medicina, Universidade de São Paulo, São Paulo, Brasil.; 2 Diretoria Regional de Brasília, Fundação Oswaldo Cruz, Brasília, Brasil.; 3 Instituto de Saúde Coletiva, Universidade Federal da Bahia, Salvador, Brasil.; 4 Departamento de Ciências da Vida, Universidade do Estado da Bahia, Salvador, Brasil.; 5 Programa de Pós-graduação em Saúde Coletiva, Universidade Católica de Santos, Santos, Brasil.

**Keywords:** HIV, Public Policies, Adolescents, Sexuality, Gender Identity, HIV, Políticas Públicas, Adolescentes, Sexualidade, Identidade de Gênero, VIH, Políticas Públicas, Adolescentes, Sexualidad, Identidad de Género

## Abstract

The United Nations has underscored the possibility of ending the HIV epidemic as a public health problem. However, an increase in the incidence among adolescents and youth has indicated a greater distance between HIV responses and the specificities of the new generations, which can maintain the epidemic for an extended period. Regards this matter, it is debated that the provision of a range of preventive methods, even if highly effective, and a conservatism that has internalized stigma within government policies, hinder the proper and essential dialogue between current preventive policies and the needs of the new generations. These generations are marked by a social representation of AIDS as a mild disease, by new gender and sexuality performances, and by the search for a more critical role in affective and sexual encounters, which includes frequent use of dating apps and substances. The hierarchy of the delivery of prevention methods is presented as a proposal for a new policy, prioritizing pre-exposure prophylaxis (PrEP) and addressing the social determinants of the HIV epidemic, including strategies to mitigate stigma. The importance of the participation of adolescents and youth in constructing the policy and the need for an intersectoral response are also reinforced.

## Resumo

A Organização das Nações Unidas tem destacado a possibilidade de eliminar a epidemia de HIV como um problema de saúde pública. Porém, um aumento da incidência em adolescentes e jovens tem indicado um maior distanciamento entre a resposta ao HIV e as especificidades próprias das novas gerações, que pode estender a epidemia por um longo período. Frente a isso, é discutido que a oferta de uma cesta universal de métodos preventivos, mesmo que altamente eficazes, e um conservadorismo que internalizou o estigma em políticas governamentais, inviabilizam o adequado e necessário diálogo entre as atuais políticas preventivas e as necessidades das novas gerações. Estas gerações que são marcadas por uma representação social da aids com menor gravidade; novas performances de gênero e de orientação sexual; e a busca de um maior protagonismo nas interações afetivas e sexuais, o que inclui o uso mais frequente de aplicativos de encontro de parcerias e de substâncias na cena de sexo. É apresentado como proposta de uma nova política a hierarquização da oferta de métodos preventivos, com a priorização da profilaxia pré-exposição (PrEP) e o enfrentamento dos determinantes sociais da epidemia do HIV, incluindo estratégias de redução do estigma. Reforça-se, ainda, a importância da participação de adolescentes e jovens na construção da política e a necessidade de uma resposta intersetorial.

## Resumen

Las Naciones Unidas señalan la posibilidad de eliminar la epidemia del VIH como un problema de salud pública. Sin embargo, un incremento de la incidencia de esta enfermedad en adolescentes y jóvenes muestra una mayor distancia entre la respuesta al VIH y las especificidades de las nuevas generaciones, lo que puede extender la epidemia durante un largo periodo. En este contexto, se discute que la oferta de una canasta universal de métodos preventivos, aunque altamente efectivos, y que un conservadurismo que interiorizó el estigma en las políticas gubernamentales hacen inviable un adecuado y necesario diálogo entre las políticas preventivas actuales y las necesidades de las nuevas generaciones. Estas generaciones están marcadas por una representación social del sida con menos gravedad, por nuevas actuaciones de género y orientación sexual y por la búsqueda de un mayor protagonismo en las interacciones afectivas y sexuales, que incluye el uso más frecuente de aplicaciones para encontrar parejas y de sustancias en la escena sexual. Se presenta como propuesta de una nueva política la priorización de la oferta de métodos preventivos, con la priorización de la profilaxis preexposición (PrEP) y la confrontación de los determinantes sociales de la epidemia del VIH, que incluyen estrategias para reducir el estigma. También refuerza la importancia de la participación de los adolescentes y jóvenes en la construcción de la política y la necesidad de una respuesta intersectorial.

## The HIV epidemic in new generations

The resurgence of the HIV epidemic in the new generations ^1^ poses a mandatory question: are current prevention policies enough to reverse this trend? Or will it be necessary to collaboratively build other bases and strategies with society to face this new scenario?

Changes in how new generations establish their affective and sexual relationships, associated with the emergence of more effective preventive methods, point to the necessary reformulation of policies without forgetting lessons learned over the last 40 years. This need for reformulation may be urgent to seize the timely opportunity to avoid a higher incidence of infection than in the previous four decades.

Adolescents and youth have been particularly affected by the HIV epidemic. The Joint United Nations Program on HIV/AIDS (UNAIDS) estimates that 28% of new infections in the world in 2019 alone occurred in the 15-24-year age group ^2^. This pattern is similar in Brazil, where 25% of infections were diagnosed in people aged 15-24 in the 2010s decade ^3^.

Since the 1980s, this increased vulnerability of adolescents has been attributed to the specificities of this life cycle phase, marked by the beginning of sexual relations in a context of biological, social, psychological, and structural transition. It has also been associated with establishing sexual partnership networks that include stable, casual, and intergenerational relationships, often associated with increased drug and alcohol consumption ^4^ ^,^ ^5^. These aspects have been worsened by insufficient access to comprehensive health care ^6^ and by interactions between social markers of differences, such as sexual and gender identity, social class, and race ^7^ ^,^ ^8^. These markers are essential to understanding the epidemic because they determine how HIV risk unevenlly affects different population groups. Socially vulnerable adolescents and youth, especially LGBTQIA+ and black people, have been disproportionately affected by the HIV epidemic than the general population, primarily due to a higher burden of violence, prejudice, and stigma since childhood ^8^. There is an even more significant HIV infection burden on transgender women, whose recognition has been hindered by wrong classifications, including epidemiological records of cases classified with cisgender men in the “homosexual relationships” transmission category ^9^.

However, the resurgence of the HIV incidence pattern draws attention to aspects beyond the specificities of adolescents and youth and their relationship with the current HIV response. Value and behaviour changes notably mark a new generation about their sexuality, such as the re-signification of gender binary ^10^ and the adoption of contemporary sexual and affective arrangements ^11^, including information technology for sexual encounters ^12^ and the use of substances for sex ^13^. Understanding these aspects is absolutely relevant. They mark, in a more pronounced way, a change in current standards, through the leading role of new generations in the search for greater identity recognition and LGBTQIA+ citizenship, at a time when Brazil is experiencing a period of strengthening policies for social participation in decisions, social inclusion and reduction of inequalities.

**Figure 1:**
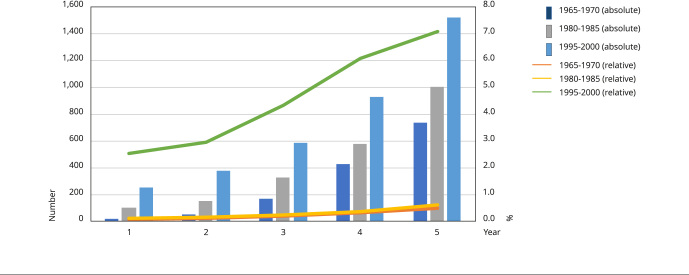
Number * and percentage contribution ** of generations in the total number of AIDS cases registered in people aged ≥ 14 years, Brazil ***.

The first evidence of this generational change is shown in the analysis of the historical series of AIDS cases in three birth cohorts in Brazil, considering the first five years of case occurrence in each cohort (Figure 1). The relative numbers of cases in the generations born between 1995 and 2000 - who, in principle, began their sexual life after introduction of antiretrovirals and their beneficial effects - surpass those recorded in previous generations, such as those born between 1965 and 1970, who began sexual relations during the emergence of the HIV epidemic and were responsible for the largest number of cases recorded in the country since the start of the disease - around 17% of the total. Furthermore, the comparison of generations born in 1995-2000 with generations born in 1965-1970 and 1980-1985 (the age that started their sexual relations during the most acute period of the epidemic, when there was no effective treatment) shows that younger generations have reversed the downward trend in the number of cases. Thus, if this trend is maintained in the coming years, the magnitude of the epidemic will be more intense in the future than it was in the last 40 years.

* Absolute number recorded in the first five years for each generation; ** Percentage in relation to the total number of cases registered in the corresponding years; *** Cases registered by the Brazilian Ministry of Health between 1980 and 2021.

## Risk compensation and new contexts of affective and sexual relationships

Changes in epidemic patterns in new generations have often been related to a risk compensation effect due to the perception of reduced disease severity with the use of antiretroviral medications to treat HIV infection. These changes would have contributed to reshaping the social representation of the disease, with the society no longer living with “fatal”, “dangerous”, and highly stigmatizing AIDS that “grew out of control” ^14^.

Evidence of risk compensation has been reported ^15^, pointing to at least two dimensions: the intention (or practice) of not having protected sexual relations and a lower prioritization of AIDS in health policies and social mobilization.

However, if the evidence seems consolidated, risk compensation’s actual contribution to understanding the recent resurgence of the epidemic in the new generations requires further analysis. Health practice, including HIV-related, has shown that the fear of getting sick is not enough to encourage social groups to adopt long-term measures that will significantly restrict their daily life and way of living. Over time, collective learning on how to live and deal with diseases and their consequences tends to grow, creating a balance between everyday needs and the risk of getting sick. This reconnection with “normality” that arises from a collective learning process does not necessarily imply an increased risk of disease as long as the new preventive strategies are adequately safe.

In the context of HIV, with effects in essential dimensions of life, these lessons learned mostly through community arrangements resulted in preventive strategies that ensured a gradual resumption of pleasure, affection, and sex even in the early years of the epidemic instead of the more restrictive recommendations of abstinence and reduced number of partners. The repertoires of sexual practices without anal or vaginal penetration, the use of condoms, and seroadaptive strategies are highlighted here ^16^ ^,^ ^17^.

Thus, after 40 years of the epidemic, we could expect that the most effective treatments, which have increased the life expectancy of people living with HIV to levels close to that of the general population, would translate into the search for a better balance between preventive strategies and different needs to perform gender and exercise sexuality.

A reflection is necessary: would preventive strategies highlighting the negative aspects of HIV treatment be relevant to encourage preventive practices? Experience indicates the possibility of a more negative than positive effect of this choice because it emphasizes a partial view of treatment and goes against the virtuous cycle that can eliminate HIV as a public health problem: diagnosis, treatment, viral suppression, and reduced HIV mortality and transmission.

In addition, despite the milder representation of the disease, qualitative studies showed that adolescents and adults at higher risk are constantly concerned about HIV infection. This can be observed in adult post-exposure prophylaxis (PEP) users, who reported significant distress while accessing and using this prophylaxis, mainly due to the fear of being infected and the anticipation of AIDS-related stigma ^18^. Similar feelings were reported by adult men who have sex with men (MSM) when searching for pre-exposure prophylaxis (PrEP). They highlighted that AIDS- and sexuality-related stigmas are barriers to seeking care ^19^, which turned into relief and increased pleasure as they felt protected with the prophylaxis ^20^. Adolescent cisgender MSM and transgender women (TGW) also showed concerns about not getting infected with HIV due to the negative consequences associated with AIDS, notably the stigma ^21^.

Understanding the search for balance between daily needs and prevention strategies in a context where AIDS is perceived with less severity than 40 years ago, may be the key to understanding the resurge of the epidemic in the new generations. From this perspective, revisiting the late 1990s and early 2000s can be enlightening ^10^. During this period, an inflection on the gender binary became more socially visible, highlighting multiple identities and possibilities between the male and female domains. This occurred concomitantly with expanding the public space for LGBTQIA+ issues through reconfiguring social identity movements based on gender and sexual orientation. The result is a context created in the early decades beginning of the 2000s, during which the recognition of gender and sexual diversity by the state and society increased.

In the HIV response, this process is especially marked by three detachments. The first is the detachment of the LGBT movement in the field of HIV, which has rearticulated itself after two decades around new social organizations, with a more specific agenda to guarantee equal rights to the general population and to implement policies to reduce sexual orientation-related violence ^22^. This also marks increased “serophobia” or “HIV-phobia” within the gay movement, somehow mirroring a sense of community. Another critical detachment is being vocal about the specificities of different gender identities and sexual orientations that would no longer be covered by a particular “gay movement umbrella”. This occurs due to stronger agendas claimed by transgender, lesbian, and asexual groups, among others, reflecting an increased number of letters to the acronym “LGBT” and a greater social visibility of these expressions ^10^. The third detachment is new forms of social organization and political practices, different from those identified in traditional nongovernmental organizations (NGOs). The so-called “collectives” appear as more fluid and less institutionalized community expressions that articulate different agendas to respond to problems and establish new experiences and practices to affirm gender and sexual identities.

This entire process is reflected in reconfiguring community living spaces and LGBTQIA+ social interactions, among other aspects. This movement expands borders and acquires new formats, with contexts more favorable to pluralities and new affective and sexual dispositions. New spaces open to the diversity of genders identities and sexual orientations have emerged in the outskirts of large urban centers, places previously marked by extreme violence against gays, with their own cultures and independent of the spaces identified as classic homoerotic spaces that were until then restricted to central regions ^23^.

The reconfiguration of interaction spaces was also influenced by different factors, especially the new communication technologies, which drastically changed the format of sexual and affective dates, and which are now mediated by apps and social networks ^12^. These new types of interaction promoted, simplified, and expanded networks and possibilities for finding partnerships, dissociating partnership interactions from physical spaces such as squares, streets, and other places where LGBTQIA+ interactions traditionally occurred. These apps are so critical in everyday life that they have become the primary means of finding sexual partners ^12^ ^,^ ^24^, with more than 60% of MSM, especially young men, reporting making use of apps once or more times a day ^12^, not only to find a casual partner but also to establish affective relationships, meet and interact with friends, and obtain different types of services or products, including commercial sex and psychoactive substances for sex. Thus, it seems natural to define apps as the “new public square” for homoerotic interactions.

Another aspect leading to essential changes in the context of sexual relations and HIV infection risk was the increased substance use in the sex scenario to facilitate interactions, improve the sexual experience, and prolong its duration ^13^ ^,^ ^25^. Although substance use in sex is not new in LGBTQIA+ environments, it has changed in recent years with chemsex, which defines long sex parties involving different partners. This scenario includes “new substances”, some synthetic and injectable substances used alone or combined and are highly addictive. The main substances used in LGBTQIA+ environments are crystal methamphetamine, gamma-butyrolactone (GBL) or gamma-hydroxybutyrate (GHB), mephedrone, and ketamine, in addition to cocaine, poppers, and marijuana. This increased use of substances in sex is attributed to internalized stigmas of gender, sexual orientation, and AIDS and the greater dissemination of dating apps ^26^. In these apps, it has been common practice to use emojis to indicate substance trade and the preference for using them at parties, on dates for two, and/or in the injectable form. The frequency of use has varied significantly by city and region, with a literature review ^25^ reporting lifetime use ranging from 3-31%. Injectable use was reported by 10% among MSM in Australia, and 16% of MSM had clinical records of complications associated with injectable substances in England ^25^.

These reconfigured sexual interaction spaces have been associated with an increased risk of sexually transmitted infection (STI), HIV, and hepatitis. This association is more evident regarding the use of apps due to the increased number of partners, unprotected sexual practices, and STIs, especially syphilis, gonorrhea, and chlamydia ^24^ ^,^ ^27^ ^,^ ^28^. The use of substances in sex also extends this evidence to HIV infection and hepatitis ^13^. The increased risk of HIV infection with apps still requires further investigation. A meta-analysis found similar HIV prevalence between application users and non-users ^28^ ^,^ ^29^. A study on adolescent MSM in Salvador (Brazil) found a borderline association between the use of apps and HIV ^30^.

At this point, it is worth reflecting again: what is the possibility of policies, strategies, and prevention methods consolidated over the last 40 years to effectively dialogue with this new context of affective and sexual interactions, which, in theory, favors practices associated with higher circulation of HIV, STIs, and hepatitis? We must consider that these policies, strategies, and methods were built in a scenario perceived as more acceptable for social groups to renounce their daily practices and experiences to ensure health and life.

## Preventive policies and methods

The concept of combination prevention emerged globally in the early 2000s and, despite being relatively controversial, established that greater sustainability and impact required prevention policies to be based on human rights, scientific evidence, community belonging, and articulation of biomedical, behavioral, and structural interventions focused on the needs of people and social groups affected by the epidemic ^17^. This concept was supported by the successful experience of countries that effectively responded to the epidemic, such as Brazil, Thailand, and Uganda.

Preventive methods based on antiretroviral medications emerged around the 2010s. However, they were emphasized as biomedical interventions of the combination prevention strategy. They started to be included in a comprehensive package of preventive measures based on the available evidence of their efficacy and/or effectiveness.

The strength of this novelty and its hypothetical potential to reduce the incidence of HIV proposed a new preventive policy paradigm, which can be summarized in two premises: there is a preventive method for each point in the chain of HIV infection (e.g., PrEP before exposure, condoms during sex, PEP after unprotected sex, and antiretroviral treatment in case of infection) and a method for each person, which must be chosen according to their needs and context. This new paradigm has often been associated with the “medicalization” of the HIV response, weakening a response based on human rights and addressing structural aspects.

Over time, programs and services have focused on delivering a comprehensive package of preventive methods showed limitations and a tendency for reduced effectiveness. The first limitation is that a basic package consisting almost exclusively of evidence produced in clinical trials generates competition between methods requiring the same structure. This would be fine if the population impact and uptake of these methods were similar or if they were widely available. A good example is PEP, which has a high degree of efficacy and low potential to prevent cases ^31^. However, it becomes a priority due to the urgency of initiating its use, which limits the capacity of services to provide PrEP. This is a priority inversion, considering that PrEP has a greater degree of reducing incidence.

Additionally, by equating all methods within a basic package, the new policy reduces its capacity to break with the mainstream approach based on condom supply. This and other aspects make it difficult for different actors to understand the more significant potential of new methods to minimize factors that hinder the use of more effective preventive methods in daily sexual interactions, such as substance and alcohol use in the sex scenario, power dynamics for negotiating preventive strategies, and affectivity in stable relationships. One of the consequences is the maintenance of a condom culture as the only (or primary) form of prevention and of reduced knowledge and/or mistrust of other preventive methods, as shown by a qualitative study with LGBTQIA+ adolescents ^32^. This is a scenario in which the new generations resemble their parents and grandparents about prevention. However, this is not true for how they perform gender, sexual orientation, and sexual and affective practices, which disconnects the current context of sexual relations from the current prevention policies.

Another aspect is that by focusing prevention on the delivery of methods, a large part of the responsibility for seeking and using it falls on the individual, who must exercise their “freedom of choice” based on information such as effectiveness, preference, and convenience. It is a kind of transposition of liberalism and its conception of the individual in health care. According to Foucault ^33^, liberalism lends rationality to the market and all relationships that permeate society. As a result, different social determinants that condition preventive practice and the HIV epidemic do not receive due importance. A significant adverse consequence is that introducing new methods within this context increases inequities and reduces the chance for populations more affected by the epidemic to benefit from the innovations promptly.

## An HIV response bringing together “new” and “old” prevention

The accumulated experience and evidence produced in recent years helped overcome the notion of combination prevention centered on a comprehensive package of methods. They can also help increase the effectiveness of policies by incorporating a less severe social representation of AIDS into their design and a leading role of the new generations in a new context of sexual interaction.

An essential first step consists of organizing delivery and demand creation strategies for prevention methods based on parameters that articulate public health impact and the promotion of human rights and equity. This would necessarily lead to setting a list of priorities to provide prevention methods, emphasizing those with the most significant potential to reduce infection incidence while tackling the needs and daily lives of populations more vulnerable to HIV.

There is no doubt that with these parameters, PrEP would be the priority method, not only because it is strongly associated with a significant reduction in HIV incidence, reaching median population coverage rates ^34^, but also because it is the method that best allows separating its utilization (taking the pill) from the sex encounter and that interferes the least with sexual interaction and practices, regardless of the context and situation in which they take place ^20^ ^,^ ^21^.

This prioritization implies explaining that PrEP should be the method of choice for populations more vulnerable to HIV, with other methods being optional for people with a lower risk of HIV exposure or who do not want, are unable, or fail to use PrEP. This PrEP prioritization becomes more critical considering that the newly available schemes, notably the long-term ones, are injectable or are in the form of vaginal rings, facilitating its use and being more convenient ^35^.

Daily oral PrEP has been less effective in adolescents than adults due to poorer adherence and persistence. Different factors may be associated with this, such as more incredible difficulty in organizing daily use, frequent changes in sexual life, and structural aspects that hinder regular clinical follow-up. Therefore, strategies that simplify and make this method fit more efficiently in the daily life of adolescents can make PrEP more meaningful for this population, including the use of telehealth, provision of the method in the community, peer-led prescription etc. ^36^.

A policy based on the rationale of a hierarchy of methods to be provided may be considered excessively normative, which disregards the diversity of people and the right to choose, and can be an excessive medicalization of prevention and/or a return to the strategy of promoting a single method, disregarding different methods such as condom use. However, it is not the absence or presence of a hierarchy that determines the degree of the normativity of a public health policy, but rather the persistence of an approach that disregards the psychosocial dimensions of sex and care, neglecting the fight against structural determinants of the epidemic, the absence of collective policy construction, limited access to all methods, and restricted information for timely decision-making.

Moreover, prevention policies must reach the necessary balance between the hierarchical provision of preventive methods and actions to face social determinants, the violation of rights, prejudice, and the stigma that restricts the right and access to prevention. In this sense, evidence has shown that structural actions and the promotion of human rights can be more effective in reducing the incidence of HIV than the emphasis on providing methods, however effective they may be ^31^. These actions would help redefine the original concept of combination prevention as the structuring basis for policies and prevention access.

In addition, a successful HIV response, among other aspects, is directly associated with the degree of societal mobilization and with an intersectoral response ^17^. This was one of the landmarks of Brazilian politics in the first decades of the epidemic; however, growing conservatism and lower visibility of AIDS as a public health problem have, in recent years, restricted the involvement of different sectors of society and weakened the health-related response to HIV.

The dispute and tension promoted by conservative sectors and values resulted in a visible internalized stigma in government policies, reducing the budget of anti-HIV projects and inhibiting essential actions in the HIV response. In the health field, for example, the lack of massive federal campaigns and strategies for populations more affected by the infection has lasted more than 20 years. The last campaign explicitly directed at MSM was in 2008, at sex workers and TGW in 2013, and at drug users in 2008 ^37^.

Stigmatization processes should be understood as social and political processes mediated by power relations and implemented by social actors to legitimize the status of domination of those in a hegemonic position ^38^. When embedded in public policies, they increase the production and reproduction of structural inequalities, reinforcing social exclusion in different contexts. This is the greatest challenge in facing the HIV epidemic and the main barrier to promoting health in populations more vulnerable to infection.

Also, in intersectoral response and considering education as one of the most relevant areas, research by the Brazilian Ministry of Health and the Brazilian Institute of Geography and Statistics (IBGE) from 2009-2015 showed a reduced number of adolescents who had sex education activities in schools, including access to condoms and prevention of unwanted pregnancy ^39^. This created a sharp contrast: the existence of more effective preventive methods coexists with generations that systematically fail to develop skills to deal with the epidemic and with prevention, perpetuating a greater fragility to control or eliminate the epidemic as a public health problem for decades. This situation was worsened by the compulsory closure of schools and teaching interruption during the COVID-19 pandemic and needs to be understood as part of the programmatic response to any public policy aimed at adolescents and youth.

Another important aspect of constructing a policy aimed at adolescents is recognizing and acting on how adult-adolescent-youth power relations are crystallized in social practices. By coining the term adult centrism, the sociology of childhood examines the distribution of power between adults in society. In the words of Fulvia Rosenberg ^40^ (p. 25), “*in the adult-centered society, the children are not. They are becoming. Their individuality ceases to exist. They are potentiality and promise*”. This leads to the question: to what extent are adolescents and young adults seen for what they “will become” in everyday educational practices, in service provision, in the formulation of public policies, and even in sexual interactions and behavior? We live in an eternal ambiguity which, on the one hand, presents a strong criticism of the adult-centric perspective to recognize the specificity of adolescents and youth as rights holders. On the other hand, this presents a set of practices that restricts the participation and engagement of adolescents and youth in identifying and building responses to the problems they experience and, above all, to the future they aspire to achieve. Thus, overcoming obstacles related to the absence of adolescents and youth in decision-making spaces will be the turning point to constructing policies to combat HIV that make sense and can reach good results with this population.

We are living in a crucial moment in the HIV epidemic. The United Nations has set a goal of ending HIV as a public health problem by 2030. However, the increased incidence in adolescents and youth that comes along with generations with less ability to deal with the epidemic points to a persistent incidence in the coming years, which will not allow the achievement of this goal ^41^. If country responses choose to eliminate HIV, we have to remember that these new preventive methods can only be a unique opportunity if they are combined with a policy that provides the best conditions for comprehensive care and HIV prevention and that responds to the needs of the new generations. It will be necessary to definitively break with the notion of AIDS constructed over the last 40 years, both from the point of view of its severity and of the supposed effectiveness in providing a comprehensive package of preventive methods. At the same time, we should bear in mind that the successful results achieved regarding the HIV response so far are the construction of a participatory policy - “with” young people instead of “about” young people - that was based on protecting and promoting human rights and on reducing vulnerabilities and inequities.
